# Broad Phenotypes of Disorders/Differences of Sex Development in *MAMLD1* Patients Through Oligogenic Disease

**DOI:** 10.3389/fgene.2019.00746

**Published:** 2019-08-29

**Authors:** Christa E. Flück, Laura Audí, Mónica Fernández-Cancio, Kay-Sara Sauter, Idoia Martinez de LaPiscina, Luis Castaño, Isabel Esteva, Núria Camats

**Affiliations:** ^1^Pediatric Endocrinology, Diabetology and Metabolism, Department of Pediatrics and Department of BioMedical Research, Bern University Hospital and University of Bern, Bern, Switzerland; ^2^Growth and Development Research Unit, Vall d’Hebron Research Institute (VHIR), Center for Biomedical Research on Rare Diseases (CIBERER), Instituto de Salud Carlos III, Barcelona, Spain; ^3^Endocrinology and Diabetes Research Group, BioCruces Bizkaia Health Research Institute, Cruces University Hospital, CIBERDEM, CIBERER, University of the Basque Country (UPV-EHU), Barakaldo, Spain; ^4^Pediatric Endocrinology Section, Cruces University Hospital, Endocrinology and Diabetes Research Group, BioCruces Bizkaia Health Research Institute, CIBERDEM, CIBERER, University of the Basque Country (UPV-EHU), Barakaldo, Spain; ^5^Endocrinology Section, Gender Identity Unit, Regional University Hospital of Malaga, Málaga, Spain

**Keywords:** whole exome sequencing, *MAMLD1*, disorders/differences of sex development, hypospadias, phenotype variability, oligogenic disorder

## Abstract

Disorders/differences of sex development (DSD) are the result of a discordance between chromosomal, gonadal, and genital sex. DSD may be due to mutations in any of the genes involved in sex determination and development in general, as well as gonadal and/or genital development specifically. *MAMLD1* is one of the recognized DSD genes. However, its role is controversial as some *MAMLD1* variants are present in normal individuals, several *MAMLD1* mutations have wild-type activity in functional studies, and the *Mamld1*-knockout male mouse presents with normal genitalia and reproduction. We previously tested nine *MAMLD1* variants detected in nine 46,XY DSD patients with broad phenotypes for their functional activity, but none of the mutants, except truncated L210X, had diminished transcriptional activity on known target promoters *CYP17A1* and *HES3*. In addition, protein expression of *MAMLD1* variants was similar to wild-type, except for the truncated L210X. We hypothesized that *MAMLD1* variants may not be sufficient to explain the phenotype in 46,XY DSD individuals, and that further genetic studies should be performed to search for additional hits explaining the broad phenotypes. We therefore performed whole exome sequencing (WES) in seven of these 46,XY patients with DSD and in one 46,XX patient with ovarian insufficiency, who all carried *MAMLD1* variants. WES data were filtered by an algorithm including disease-tailored lists of *MAMLD1*-related and DSD-related genes. Fifty-five potentially deleterious variants in 41 genes were identified; 16/55 variants were reported in genes in association with hypospadias, 8/55 with cryptorchidism, 5/55 with micropenis, and 13/55 were described in relation with female sex development. Patients carried 1-16 variants in 1-16 genes together with their *MAMLD1* variation. Network analysis of the identified genes revealed that 23 genes presented gene/protein interactions with MAMLD1. Thus, our study shows that the broad phenotypes of individual DSD might involve multiple genetic variations contributing towards the complex network of sexual development.

## Introduction

Disorders/differences of sex development (DSD) occur when there is a discordance between chromosomal, gonadal, and genital sex (Ostrer, 2014). DSD may be due to mutations in any of the genes involved in sex determination and development in general, as well as gonadal and/or genital development specifically (Ostrer, 2014).

*MAMLD1* (Xq28, OMIM 300120) is one of the recognized DSD-related genes (Fukami et al., 2006; Baxter et al., 2015). Variations in *MAMLD1* sequence have been described mainly in 46,XY DSD individuals, mostly associated with hypospadias (Fukami et al., 2006; Kalfa et al., 2008; Chen et al., 2010; Kalfa et al., 2012; Metwalley and Farghaly, 2012; Camats et al., 2015; Igarashi et al., 2015; Eggers et al., 2016), but also with other DSD phenotypes, including micropenis (Chen et al., 2010; Kalfa et al., 2012; Camats et al., 2015; Liu et al., 2017), and/or crypthorchidism (Fukami et al., 2006; Kalfa et al., 2008; Kalfa et al., 2012; Camats et al., 2015), 46,XY with female external genitalia (Fukami et al., 2006; Camats et al., 2015) and 46,XY with complete gonadal dysgenesis (Ruiz-Arana et al., 2015). Furthermore, one homozygous *MAMLD1* variant was also reported in a 46,XX patient with gonadal dysgenesis, primary amenorrhea, bilateral streak gonads and clitoromegaly (Brandao et al., 2011).

However, the role of MAMLD1 in sex development is controversial for several reasons: a) some *MAMLD1* variants are present in the normal population (Fukami et al., 2006; Chen et al., 2010; Gaspari et al., 2011; Kalfa et al., 2011); b) the same *MAMLD1* variant may be present in patients with different phenotypes (Camats et al., 2015); c) *MAMLD1* variants are not present in all DSD individuals of the same family (Fukami et al., 2006); d) several *MAMLD1* mutations present wild-type activity in functional studies (Camats et al., 2015); and e) the *Mamld1*-knockout male mouse presents with normal genitalia and reproduction (Miyado et al., 2012; Miyado et al., 2017).

*MAMLD1* is expressed in human fetal and adult testis and in human ovaries (Fukami et al., 2006; O’Shaughnessy et al., 2007; Camats et al., 2015), and seems to be involved in sex development in fetal life and in adult reproductive function. Yet its exact role is not clear. It is expressed in mice gonadal cells during start of androgen biosynthesis up to male external genitalia formation and is therefore thought to be involved in the expression of Leydig-cell genes (Miyado et al., 2012), as well as supporting testosterone production in critical periods of male development (Fukami et al., 2008; Nakamura et al., 2011). In contrast, *Mamld1*-KO mice present normal external genitalia (but small testes and reduced seminiferous tubule size and proliferating germ cells) and reproduce similarly to wild-type mice (Miyado et al., 2012; Miyado et al., 2017). These findings challenge the role of MAMLD1 in sex development.

In a previous study, we tested functional activity of nine *MAMLD1* variants detected in nine 46,XY DSD patients with broad phenotypes (Camats et al., 2015). None of the *MAMLD1* mutants, except truncated L210X, had diminished transcriptional activity on known target promoters *CYP17A1* and *HES3*. In addition, protein expression of *MAMLD1* variants was similar to wild-type, except for the truncated L210X. We therefore hypothesized that *MAMLD1* variants may not be sufficient to explain the phenotype in 46,XY DSD carriers, and that further genetic studies should be performed to search for additional hits explaining the broad variability.

In the past decade, High throughput sequencing (HTS) has changed the genetic approach in research and diagnostics. Whole-exome sequencing (WES) has led to the discovery of many new genes and has given insight into complex traits. Oligogenic inheritance is currently discovered for several disorders by HTS. In the field of sex development, digenic inheritance has recently been suggested in a 46,XY DSD patient with gonadal dysgenesis (*NR5A1* and *MAP3K1* variants) (Mazen et al., 2016); in a family with 46,XY DSD males (*NR5A1* variants) and 46,XX POF females (*NR5A1* and *TBX2*) (Werner et al., 2017); as well as in a DSD patient with ambiguous genitalia, micropenis, and inguinal testes (*SEMA3A* and *AKR1C4*) (Fan et al., 2017). Similarly, we found oligogenic origin of disease in heterozygous *NR5A1* 46,XY DSD patients by performing WES (Camats et al., 2018). In addition, in patients with hypospadias, an oligogenic origin was suggested by two other NGS studies (Kon et al., 2015; Eggers et al., 2016).

Therefore, in this study, we performed WES in seven 46,XY patients with DSD (Camats et al., 2015) and one 46,XX patient with ovarian insufficiency, who all carried *MAMLD1* variants. WES data were filtered by common tools and a disease-tailored algorithm including *MAMLD1*-related and DSD-related known and candidate genes. Additional hits in likely disease-causing genes were detected in all eight *MAMLD1* carriers. Our results suggest that oligogenic origin of disease may contribute towards the broad phenotypes of human *MAMLD1*.

## Patients and Methods

### Patients

The study was approved by the Ethics Committee of Hospital Universitari Vall d'Hebron (Barcelona, Spain) (CEIC: PR(IR)23/2016). Written informed consent was obtained from the patients for the publication of their cases. Eight DSD patients (seven 46,XY and one 46,XX) each carrying one *MAMLD1* variant were analyzed using WES. Clinical and genetic characteristics of 46,XY patients were previously reported in detail in Camats et al., (2015 and are summarized in **Table 1** together with the 46,XX patient.

**Table 1:** Clinical, biochemical and genetic characteristics of the studied *MAMLD1* patients.

Patient	Karyotype assigned sex	Phenotype and origin	Gonadal function (age)	Adrenal function (age)	*MAMLD1* variant	Variants after filtering by gene list (A)	Candidate variants (B)	Candidate genes (C)
1 (7)	46,XY Female	Penoscrotal hypospadias. Small penis. Unilateral cryptorchidia. Histology: normal for age (2y). Venezuelan origin.	Normal hCG test.	Normal Synacthen test.	p.V505A NM_005491:c.1514T>C	547	9	7
2 (6)	46,XY Male	Penoscrotal hypospadias. Small penis. Testes: 2 ml. Spanish origin.	Normal baseline T (3m). Normal hCG test (9m).	Normal baseline (3d).	p.A503E NM_005491:c.1508C>A	492	1	1
3 (9)	46,XY Female	Penoscrotal hypospadias. Small penis. Histology: normal for age (nests of normal Leydig cells; normal fertility index (1y) Müllerian ducts. Spanish origin	Normal baseline (12m). No hCG test.	NA	p.S730S NM_005491:c.2190G>A	570	2	2
4 (3)	46,XY Female	Female genitalia. Gonads in labia. Spanish origin.	Normal hCG test (2y).	Normal baseline (2y).	p.H347Q NM_005491:c.1041C>A *rs62641609*	633	4	4
5 (4)	46,XY Male	Penoscrotal hypospadias. Testes 2 ml. Spanish origin.	Normal hCG test. Normal AMH (2.5y).	Normal baseline (2.5y).	p.H347Q NM_005491:c.1041C>A *rs62641609*	929	6	6
6 (8)	46,XY Male	Penoscrotal hypospadias. Small penis. Testes 1 ml. Esophageal atresia. Right aortic arch. North African origin.	Normal prepubertal baseline T (15 m). Normal AMH.	Normal baseline (15 m).	p.L724V NM_005491:c.2170C>G	710	16	16
7 (5)	46,XY Male	Hypospadias. Short penis. Testes 8 ml. Delayed puberty. Gynaecomastia. Fathered a boy. Swiss origin.	Normal baseline T and gonadotropins (70y). Fathered a boy.	Normal baseline (70y).	*p.Q501Q502* NM_005491:c.1503_1504dupCAGCAG	429	5	5
8	46,XX Female	Female external genitalia. Small ovaries and uterus, with fallopian tubes. Primary amenorrhea (15y). Histology: large amount of primordial follicles (no evidence of maturation), atresic follicles. Delayed growth. Spanish origin.	High gonadotropins and low/normal estradiol (27y).	Normal (27y).	NM_005491:c.*126C>MIT	574	14	13

All patients presented one hemizygous/heterozygous variant in MAMLD1. In parentheses: patients in Camats et al. (2015); NA, not analyzed; d, day(s); m, month(s); y, year(s). (A) Filtered by DSD-related and MAMLD1-related gene list. (B) Number of candidate variants per patient: related to sex development, DSD phenotypes, and/or in MAMLD1-related genes, and with MAF ≤ 0.01. (C) Number of candidate genes per patient: genes containing at least one candidate variant per patient.

### DNA Extraction, WES and Bioinformatic Analysis

DNA was extracted from blood leukocytes using QiaCube (Qiagen, Hilden, Germany) or manually using a DNA isolation kit (Qiagen). WES was performed by CNAG (Centre Nacional d’Anàlisi Genòmica, Barcelona, Spain). Libraries were prepared with a SureSelect Human All Exon V5 capture kit (Agilent, Santa Clara, CA, USA) and sequenced with a HiSeq™ 2000 sequencing system (v3, 2x100, Illumina, San Diego, CA, USA). Putative candidate variants were confirmed by Sanger sequencing.

The genomic datasets were annotated (alignment with human genome hg19/grch37) and filtered with the functional annotation of genetic variants from HTS data (ANNOVAR; http://annovar.openbioinformatics.org/) (Wang et al., 2010), visualized and explored in Integrative Genomics Viewer (IGV, Broad Institute, Cambridge, MA, USA; https://www.broadinstitute.org/igv/ (Robinson et al., 2011). Frequencies of variants of relevant candidate genes were obtained from the Genome Aggregation Database (gnomAD; http://gnomad.broadinstitute.org/about) (Lek et al., 2016) and the Collaborative Spanish Variant Server (CSVS; CIBERER BIER, Valencia, Spain; http://csvs.babelomics.org/; August 2018) (Dopazo et al., 2016). gnomAD includes gene variants from exome and genome sequencing data: 123,136 exomes and 15,496 genomes from unrelated individuals (from population and disease-specific studies). CSVS database includes (among others) exomes from a population of 267 healthy unrelated subjects.

WES data were filtered by a disease-tailored list of *MAMLD1*-related and DSD-related known and candidate genes (n = 606) similar to the algorithm previously set up for Camats et al., (2018). We generated a project-specific filter for DSD-related and *MAMLD1*-related genes by searching in published literature and databases. DSD-related genes are part of our DSD-gene database and tools (Camats et al., 2018), which have been currently updated. The DSD-related gene list included genes with reported (potentially) deleterious variants in patients with 46,XY and 46,XX DSD, genes with reported (potentially) disease-causing variants in syndromic patients with involvement of sex development, those “related” to DSD conditions in KO/mutant animal models (mice and rats), and also overexpressed, upregulated or downregulated genes in rodent embryonic gonadal cells (Camats et al., 2018). For the search for functional human partners of MAMLD1 and for possible interactions within interesting genes, the Search Tool for the Retrieval of Interacting Genes/Proteins (STRING, http://string-db.org/) (Jensen et al., 2009) and the Biological General Repository for Interaction Datasets (BioGRID, thebiogrid.org) (Stark, 2006) were used.

We used 30 pathogenic predictors to predict possible impact of amino acid substitutions on the structure, function and evolutionary conservation of corresponding human proteins and to predict impact on splicing. These *in-silico* predictors were accessed through ANNOVAR (Wang et al., 2010) annotation and run through Alamut Visual 2.11 (https://www.interactive-biosoftware.com/es/alamut-visual/). Functional exonic predictors were CADD (Combined Annotation Dependent Depletion of single-nucleotide and insertion/deletion variants, http://cadd.gs.washington.edu/) (Kircher et al., 2014), SIFT (Scale-invariant feature transform; http://sift.jcvi.org/), PolyPhen-2 (Polymorphism Phenotyping v2: HumDiv, HumVar; http://genetics.bwh.harvard.edu/pph2/index.shtml), Provean (http://provean.jcvi.org), MutationAssessor (http://mutationassessor.org/r3/), Mutation Taster (http://www.mutationtaster.org/), LRT, FATHEMM, Fathmm-MKL, PROVEAN, VEST3 (Variant Effect Scoring Tool), MetaSVM, MetaLR, MCAP, DANN and fitCons. Exonic predictors on evolutionary conservation were: GERP++, phyloP (vertebrate and mammalians), phastCons (vertebrate and mammalians) and SiPhy. Splicing predictors were: splicing predictors from dbscSNV ADA and RF and SPIDEX splicing predictor (DPSI), and those splicing predictors from Alamut Visual software were: SSF, MaxEnt, NNSPLICE, GeneSplicer and Ex-Skip.

The following bioinformatics software tools were used for the interpretation and classification of variants: InterVar (http://wintervar.wglab.org/, clinical interpretation of genetic variants by the ACMG/AMP 2015 guideline), VarSome (The Human Genomics Search Engine; https://varsome.com/), ClinVar (https://www.ncbi.nlm.nih.gov/clinvar/) and Alamut Visual 2.11 (https://www.interactive-biosoftware.com/es/alamut-visual/). We searched for reported (potentially) disease-causing variants with the Human Gene Mutation Database (HGMD^®^ Professional 2018.2, http://www.biobase-international.com/product/hgmd; Biobase) and dbSNP (http://www.ncbi.nlm.nih.gov/snp/). We used STRING for the search for interactions within genes carriers of interesting variants (DSD-related and/or *MAMLD1*-related). Data from STRING are extracted from known interactions (curated databases, experimentally determined interactions), predicted interactions (gene neighborhood, gene fusions, gene co-occurrence) and other inferred evidences such as text mining, co-expression and protein homology. We used Pubmed (https://www.ncbi.nlm.nih.gov/pubmed/) and OMIM (https://www.omim.org) to build our DSD gene list and for further data analysis. The datasets generated for this study are publicy available in dbSNP (Sherry, 2001): https://www.ncbi.nlm.nih.gov/projects/SNP/snp_viewBatch.cgi?sbid=1063030.

### Variant Analysis Per Patient

**Figure 1:**
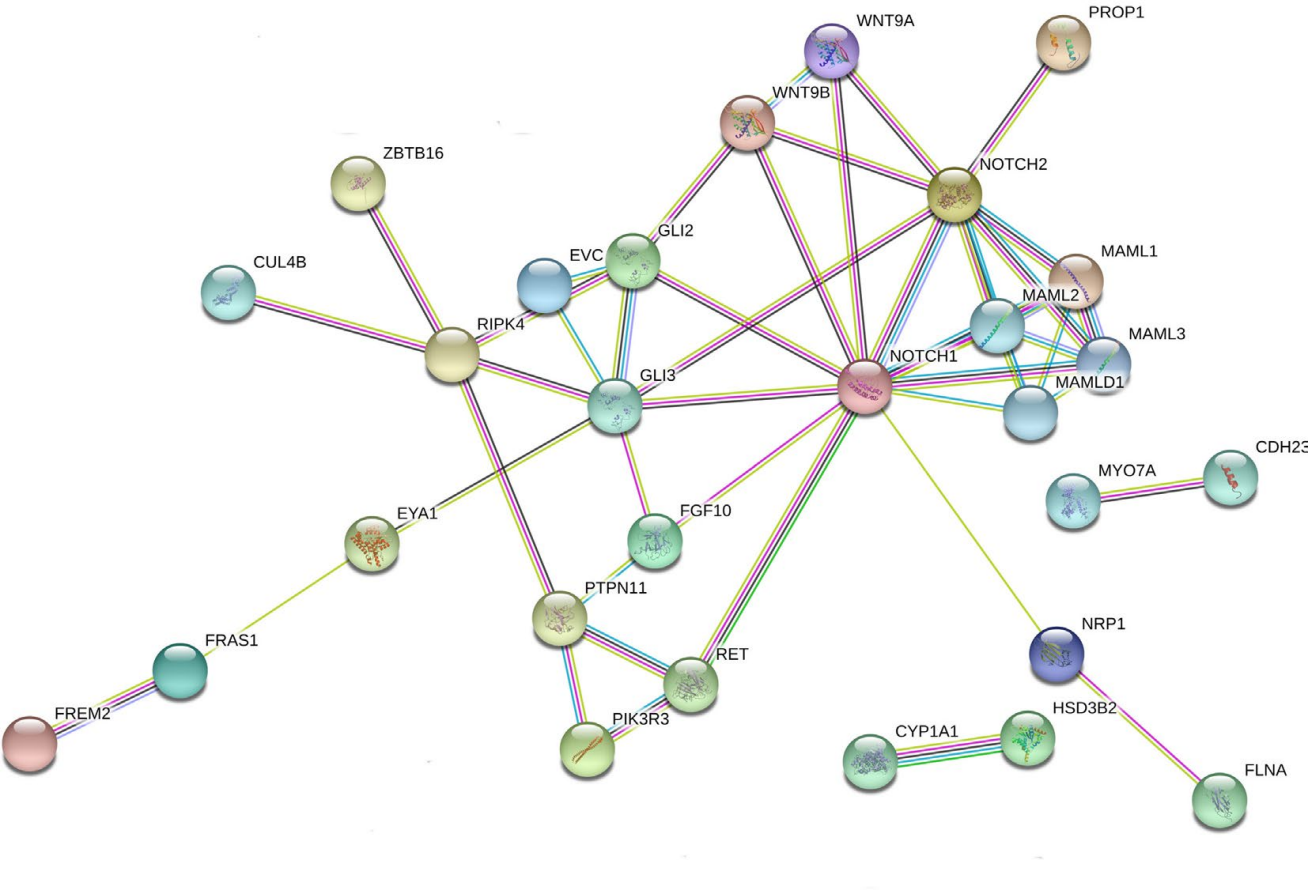
Interaction network of DSD- and *MAMLD1*-related genes identified in DSD individuals harboring genetic variants in *MAMLD1*. The scheme depicts an overview of detected genes and their interrelationship. For the search for functional human partners, the Search Tool for the Retrieval of Interacting Genes/Proteins (STRING, http://string-db.org/) was used. Nodes represent proteins. Filled nodes show proteins with known or predicted 3D structure. Empty nodes depict proteins with unknown 3D structure. Candidate genes are underlined. Known interactions correspond to curated databases (turquoise lines) and experimentally determined interactions (pink lines). Predicted interactions correspond to gene neighborhood (green lines), gene fusions (red lines) and gene co-occurrence (blue lines). Other interactions correspond to text mining (yellow lines), co-expression (black lines) and protein homology (violet lines).

**Figure 2:**
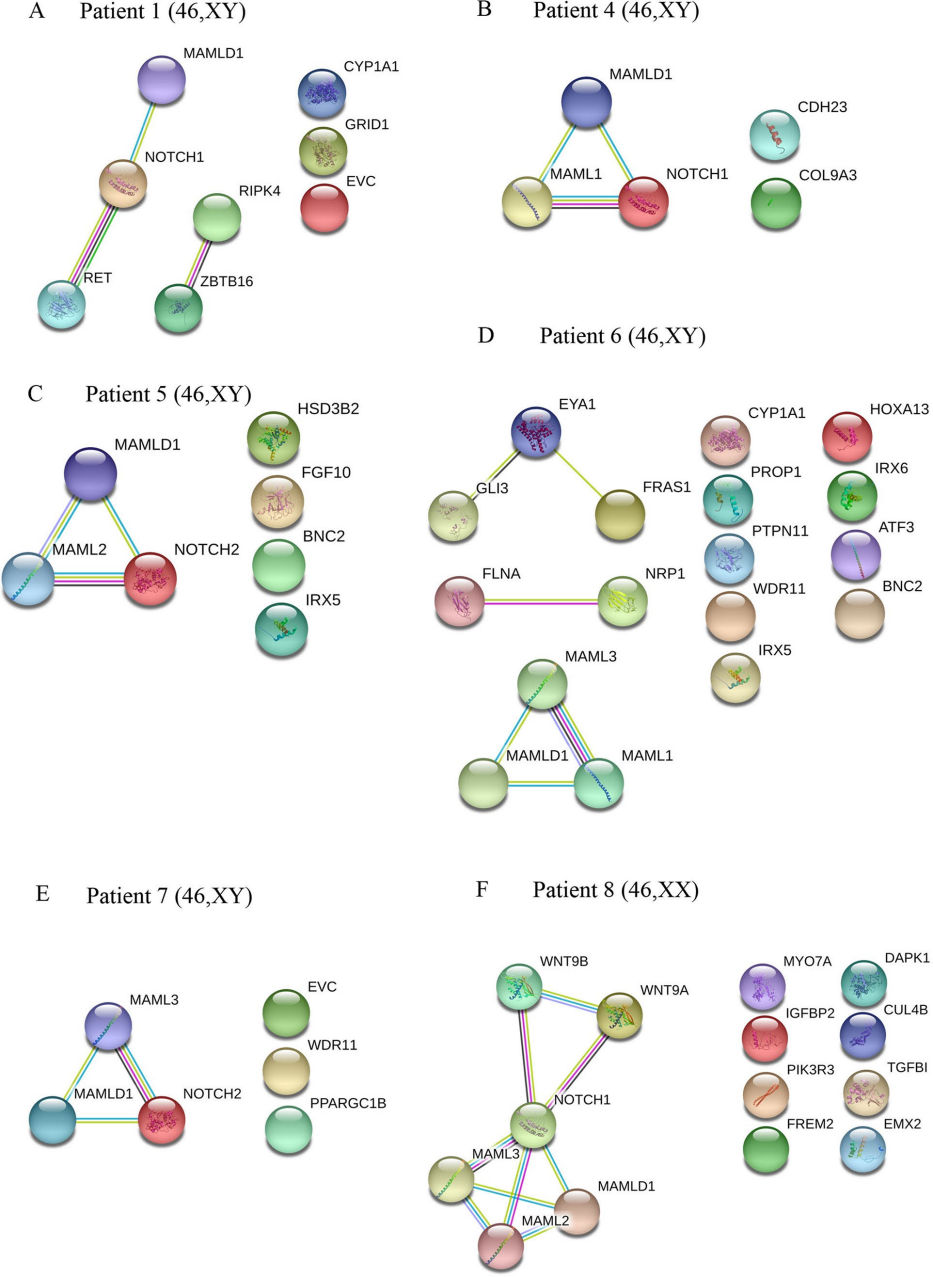
Interaction networks of DSD- and *MAMLD1*-related genes identified per single *MAMLD1* individual. **(A)** to **(F)** correspond to the interaction networks per patient. For the search for functional human partner, the Search Tool for the Retrieval of Interacting Genes/Proteins (STRING, http://string-db.org/) was used. Nodes represent proteins. Filled nodes show proteins with known or predicted 3D structure. Empty nodes depict proteins with unknown 3D structure. Candidate genes are underlined. Known interactions correspond to curated databases (turquoise lines) and experimentally determined interactions (pink lines). Predicted interactions correspond to gene neighborhood (green lines), gene fusions (red lines) and gene co-occurrence (blue lines). Other interactions correspond to text mining (yellow lines), co-expression (black lines) and protein homology (violet lines). Genes with no interactions are on the right side of each network.

After annotation, variant analysis was performed by the following steps. A) Each patient’s WES data were first filtered by our *MAMLD1*- and DSD-related known and candidate genes. B) We kept variants with MAF (minor allele frequency) ≤0.015 or not detected in gnomAD, and variants with the following predicted type, consequences and locations: splicing (intronic or exonic), exonic, intergenic, regulatory. C) We confirmed the correct annotation and location of variants by checking their alignment data in IGV (alignment with human genome hg19/grch37) (data not shown). D) We excluded variants that were considered non-relevant for our study: E.g. 1) variants found in more than two patients, 2) variants in repeat regions, 3) variants in genes or gene regions with high variability, 4) variants with low coverage and/or low quality, 5) variants with non-similar allelic depths. E) We revised variants with the annotated pathogenic predictors: functional exonic, evolutionary-conservation and splicing predictors (ANNOVAR and Alamut Visual software), as previously described. F) We run InterVar and VarSome to classify the variants, searched for reported (potentially) human disease-causing variants with the HGMD, and revised evidences of relationship with DSD, sex development and clinical phenotype of each patient with literature and database search. G) We used STRING to find out interactions among genes carriers of interesting variants (DSD-related and/or *MAMLD1*-related) (**Figures 1** and **2**). H) We checked MAF in a healthy cohort of Spanish population (CSVS: 267 unrelated healthy controls). I) We rejected variants with MAF ≥ 0.01 (gnomAD, CVSV, August 2018), thus less plausible to be a DSD-causing variant. Importantly, synonymous variants were not rejected because it has been shown that they may affect splicing.

## Results

WES performed in eight unrelated subjects (seven 46,XY and one 46,XX) harboring hemizygous/heterozygous *MAMDL1* variants revealed several candidate gene variants that potentially contribute to each patient’s phenotype. A detailed summary of patients’ characteristics and number of variants and genes is shown in **Table 1**. A list of identified candidate variants and corresponding information from literature is given in **Table 2**.

**Table 2:** Identified genes and variants per patient after specific filtering.

Patient	*Gene*	Chromosome: Coordinates	Type/consequence	HGVSc,HGVSp	dbSNP ID	gnomAD: MAF	CSVS: MAF	Predictors					Interpretation/classification (6)				Evidence	
								Exonic predictor: CADD (1)	Exonic predictors: Functional impact (2)	Exonic predictors: conservation (3)	Splicing predictor (4)	Splicing predictors (Alamut) (5)	InterVar	ClinVar	VarSome	ACMG	HGMD: variant (7)	Gene characteristics: evidences for genotype-phenotype correlation (8)
1	*CYP1A1*	15:75013544	snv/missense	NM_000499.5:c.1162C>G:p.(His388Asp)	–	ND	ND	24.9	13	6	0	5	VUS	ND	–	VUS: PM2, PP3	No	Association to hypospadias (ᴔvan der Zanden et al., 2012)
1	*EVC*	4:5798754	snv/missense	NM_153717.2:c.1892C>T:p.(Thr631Met)	rs139481521	0.0003	ND	23.5	5	3	0	3	VUS	VUS	–	VUS: BP4	No	Syndromic (Ellis–van Creveld syndrome) micropenis (D’Asdia et al., 2013; Ibarra-Ramirez et al., 2017), gonadal development? (Beverdam and Koopman, 2006; Jameson et al., 2012)
1	*EVC*	4:5785368	snv/synonymous	NM_153717.2:c.1653G>A:p.(Pro551=)	rs151293705	0.0003	ND	2.382	NA	NA	1	3	Likely benign	other	VUS	VUS: BP7	No	Syndromic (Ellis–van Creveld syndrome) micropenis (D’Asdia et al., 2013; Ibarra-Ramirez et al., 2017), gonadal development? (Beverdam and Koopman, 2006; Jameson et al., 2012)
1	*GRID1*	10:87966142	snv/missense	NM_017551.2:c.499A>G:p.(Met167Val)	rs956188880	0.00004	ND	10.04	1	5	0	NA	VUS	ND	–	VUS: BP4	No	Candidate to hypospadias (van der Zanden et al., 2012)
1	*NOTCH1*	9:139399213	snv/synonymous	NM_017617.5:c.4930C>T:p.(Leu1644=)	rs568700183	0.0003	ND	0.018	NA	NA	0	4	Likely benign	Likely benign|Likely benign	Likely benign	Likely benign: BP6, BP7	No	Related to SHH and FGF10 (Grinspon and Rey, 2014)
1	*RET*	10:43609955	snv/missense	NM_020975.5:c.1907C>T:p.(Thr636Met)	rs1035958105	0.00001	0	23.0	6	6	0	NA	VUS	VUS	VUS	Likely pathogenic: PM1, PM2, PP2, PP3	DM; thyroid carcinoma	Syndromic (CAKUT syndrome) cryptorchidism (ᴔChatterjee et al., 2012), gonadal development? (Jameson et al., 2012; Li et al., 2014)
1	*RIPK4*	21:43161468	snv/missense	NM_020639.2:c.1885G>A:p.(Asp629Asn)	rs199669994	0.00004	ND	7.899	4	4	NA	NA	VUS	ND	–	VUS: PM2 BP1	No	Syndromic (Popliteal pterygium syndrome) genital hypoplasia (Mitchell et al., 2012), micropenis, hypoplastic scrotum, inguinal hernia (Kalay et al., 2012), gonadal development? (Beverdam and Koopman, 2006; Jameson et al., 2012)
1	*RIPK4*	21:43176830	snv/missense	NM_020639.2:c.329C>T:p.(Ser110Leu)	rs200823657	0.00005	ND	22.7	3	5	0	NA	VUS	NDw	–	VUS: PM2 BP1	No	Syndromic (Popliteal pterygium syndrome) genital hypoplasia (Mitchell et al., 2012), micropenis, hypoplastic scrotum, inguinal hernia (Kalay et al., 2012), gonadal development? (Beverdam and Koopman, 2006; Jameson et al., 2012)
1	*ZBTB16*	11:114027160	snv/intronic	NM_006006.5:c.1366+4G>C	–	ND	ND	13.81	NA	NA	1	4	ND	ND	–	VUS: PM2 BP1	No	Syndromic (deletion 11q23) cryptorchidism and micropenis (Fischer et al., 2008)
2	*RECQL4*	8:145738828	snv/missense	NM_004260.3:c.2237C>T:(p.Ala746Val)	rs201883228	0.0002	ND	26.8	6	5	NA	NA	VUS	VUS	VUs	VUS: BP1	No	Syndromic (Rothmund–Thomson syndrome) hypospadias, bilateral inguinal hernia (Kellermayer et al., 2005)
3	*GLI2*	2:121747688	snv/missense	NM_005270.4:c.4198G>T:p.(Gly1400Cys)	rs143914758	0.0001	ND	23.4	7	2	NA	NA	Likely benign	ND	VUS	VUS: -	No	Increased risk of hypospadias (Carmichael et al., 2013), male gonadal development (Jameson et al., 2012), masculinization of male external genitalia (Rey et al., 2000)
3	*RECQL4*	8:145737701	snv,missense	NM_004260.3:c.3062G>A:p.(Arg1021Gln)	rs34666647	0.004		2.375	2	0	0	4	Likely benign	other|Benign	Benign	Likely benign: PM2, PM5, BP1, BP4	Cancer	Syndromic (Rothmund–Thomson Syndrome) hypospadias, bilateral inguinal hernia (Kellermayer et al., 2005)
4	*CDH23*	10:73559034	snv/nonsense	NM_022124.5:c.7221C>A:p.(Tyr2407*)	rs779038178	ND	0.002	35	5	6	1	3	Likely pathogenic	ND	–	Pathogenic: PVS1, PM2, PP3	No	Gonadal development? (Jameson et al., 2012; Li et al., 2014)
4	*COL9A3*	20:61448956	snv/missense	NM_001853.4:c.116C>G:(p.(Pro39Arg)	rs1028982816	0.00001	ND	22.7	12	5	0	NA	VUS	ND	–	VUS: PP3	No	Male gonadal development (Nef et al., 2005; Beverdam and Koopman, 2006; Jameson et al., 2012)
4	*MAML1*	5:179193385	snv/synonymous	NM_014757.4:c.1374C>T:p.(Asp458=)	rs61748799	0.003	0.004	0.089	NA	NA	0	1	Likely benign	ND	–	VUS: BP7	No	No (MAMLD1-related)
4	*NOTCH1*	9:139401803	snv/synonymous	NM_017617.5:c.3597C>T:p.(Leu1199=)	rs150666307	0.00009	ND	11.70	NA	NA	0	3	Likely benign	Likely benign	Likely benign	Likely benign: BP6, BP7	No	Related to SHH and FGF10 (Grinspon and Rey, 2014)
5	*BNC2*	9:16436324	snv/missense	NM_017637.5:c.1868C>A:p.(Pro623His)	rs114596065	0.0022	0.002	5.290	8	6	NA	NA	Benign	Benign	Benign	VUS: BP6	No	Hypospadias (Bhoj et al., 2011; van der Zanden et al., 2012; Baxter et al., 2015; Kon et al., 2015), gonadal development? (Jameson et al, 2012)
5	*FGF10*	5:44388817	snv/upstream/missense	NG_011446.1:c.-33G>A	rs17233910	0.005	0.002	19.99	NA	NA	NA	NA	ND	ND	–	VUS: -	No	Increased risk hypospadias in human (van der Zanden et al., 2012; Carmichael et al., 2013; Svechnikov et al., 2014); development of the glans penis (Rey et al., 2000)
5	*HSD3B2*	1:119964831	snv/missense	NM_000198.3:c.707T>C:p.(Leu236Ser)	rs35887327	0.003788	ND	8.277	3	3	NA	NA	Likely benign	VUS	VUS	Likely Pathogenic: PS1, PP2, PP5, BP4	DM?; HSD3B2 deficiency	Hypospadias (Codner et al., 2004; Kon et al., 2015; Eggers et al., 2016), sex development (Baxter and Vilain, 2013; Baxter et al., 2015), hormone synthesis (ᴔMcCartin et al., 2000)
5	*IRX5*	16:54967040	snv/missense	NM_005853.5:c.707C>T:p.(Pro236Leu)	rs115549200	0.0088	ND	11.97	3	4	NA	NA	Benign	ND	–	VUS: BP4	No	Association to hypospadias (Geller et al., 2014; Grinspon and Rey, 2014), female gonadal development? (Nef et al., 2005)
5	*MAML2*	11:95826473	snv/missense	NM_032427.3:c.722G>A:p.(Arg241Gln)	rs111958464	0.005	ND	32	6	6	0	NA	VUS	ND	–	VUs: -	No	No (MAMLD1-related)
5	*NOTCH2*	1:120469147	snv/nonsynonymous	NM_024408.3:c.3980A>G:p.(Asp1327Gly)	rs61752484	0.0037	0.004	20.4	6	6	0	NA	Likely benign	Benign	Benign	VUS: BP6	DM?; cardiopathy	Primary ovarian failure (Patiño et al., 2017); male gonadal development? (Jameson et al., 2012)
6	*ATF3*	1:212788544	snv/missense	NM_001674.3:c.181G>T:p.(Ala61Ser)	–	ND	ND	7.826	3	5	1	NA	Benign	ND	–	VUS: PM2, BP4	No	Hypospadias (Beleza-Meireles et al., 2008; van der Zanden et al., 2012), female gonadal development? (Jameson et al., 2012)
6	*BNC2*	9:16436324	snv/missense	NM_017637.5:c.1868C>A:p.(Pro623His)	rs114596065	0.0022	0.002	5.290	8	6	NA	NA	Benign	Benign	Benign	VUS: BP6	No	Hypospadias (Bhoj et al., 2011; van der Zanden et al., 2012; Baxter et al., 2015; Kon et al., 2015), gonadal development? (Jameson et al., 2012)
6	*CYP1A1*	15:75012979	snv/missense	NM_001319216.2:c.1303C> A:p.(Arg435Ser)	rs41279188	0.0047	ND	33	11	5	NA	NA	VUS	ND	Benign	VUS: PP3, BP6	No	Association to hypospadias (van der Zanden et al., 2012)
6	*EYA1*	8:72211882	snv/synonymous	NM_000503.5:c.630T>C:p.(Ser210=)	rs373102227	0.00008	ND	10.56	NA	NA	1	4	Likely benign	VUS	VUS	VUS: PP3, BP7	No	Associated to hypospadias (Grinspon and Rey, 2014; Hwang et al., 2014), male gonadal development? (Jameson et al., 2012)
6	*FLNA*	X:153596078	snv/synonymous	NM_001456.3:c.651C>T:p.(Asp217=)	rs34644500	0.0002	ND	5.473	NA	NA	1	3	Likely benign	Likely benign	Likely benign	Likely benign: BP4, BP6, BP7	No	Hypospadias, cryptorchidism, diminished androgen receptor (Carrera-García et al., 2017), female gonadal development? (Jameson et al., 2012)
6	*FRAS1*	4:79334181	snv/missense	NM_025074.6:c.4367T>C:p.(Ile1456Thr)	rs560902495	0.00003	ND	24.6	12	5	NA	NA	VUS	ND	–	VUS: PM2, PP3, BP1	No	Syndromic (Fraser syndrome) abnormal genitourinary system (Retterer et al., 2016; Kornacki et al., 2017); female gonadal development? (Jameson et al., 2012)
6	*GLI3*	7:42066017	snv/intronic	NM_000168.5:c.1029-6G>A	rs748670269	0.00002	ND	0.004	NA	NA	0	3	NA	ND	–	VUS: BP4	No	Increased risk of hypospadias (Carmichael et al., 2013), early genital primordia (Rey and Grinspon, 2011), female gonadal development? (Jameson et al., 2012)
6	*HOXA13*	7:27239079	snv/missense	NM_000522.4:c.618C>G:p.(Phe206Leu)	rs774388075	0.00002	ND	22.3	5	5	NA	NA	VUS	ND	–	VUS: -	No	Associated to hypospadias (Beleza-Meireles et al., 2007; van der Zanden et al., 2012; syndromic, Hand-foot-genital)/Guttmacher syndrome) hypospadias (Innis et al., 2002), small penis (Goodman et al., 2000), genital tubercle development (Grinspon and Rey, 2014)
6	*IRX5*	16:54965347	deletion/inframe	NM_005853.5:c.240_242delCTC:p.(Ser81del)	rs1057518726	ND	ND	–	NA	NA	NA	NA	NA	VUS	VUS	VUS: PM4: nonframeshift deletion	No	Association to hypospadias (Geller et al., 2014; Grinspon and Rey, 2014), female gonadal development? (Nef et al., 2005)
6	*IRX6*	16:55362842	snv/missense	NM_024335.2:c.952T>A:p.(Phe318Ile)	rs61743419	0.0014	ND	5.599	3	4	0	NA	VUS	ND	–	VUS: -	No entry for this gene	Associated to hypospadias (Grinspon and Rey, 2014)
6	*MAML1*	5:179193168	snv/missense	NM_014757.4:c.1157G>T:p.(Gly386Val)	rs777367230	0.0001	0.003	22.9	9	5	0	NA	VUS	ND	–	VUS: -	No	No (MAMLD1-related)
6	*MAML3*	4:140811687 NOT CONFIRMED	snv/synonymous	NM_018717.5:c.903C>T:p.(Asp301=)	rs76066862	0.0015	0.002	2.445	NA	NA	NA	2	Likely benign	ND	–	VUS: -	No	Female gonadal development? (Jameson et al., 2012)
6	*NRP1*	10:33469272	snv/missense	NM_003873.5:c.2504G>A:p.(Gly835Asp)	–	ND	ND	5.556	6	4	NA	NA	VUS	ND	–	VUS: PM2	No	DSD (Baxter et al., 2015), gonadal development? (Jameson et al., 2012)
6	*PROP1*	5:177421299	deletion/frameshift	NM_006261.4:c.150delA:p.(Arg53Aspfs*112)	rs587776683	ND	ND	–	NA	NA	NA	NA	NA	Pathogenic	Likely pathogenic	VUS: PVS1, PP5	DM; pituitary hormone deficiency	No, hypogonadotropic hipogonadism (Reynaud et al., 2005; Reynaud et al., 2005; Baxter and Vilain, 2013; Baxter et al., 2015; Eggers et al., 2016)
6	*PTPN11*	12:112856827	snv/upstream	NM_002834.4:c.-89G>A	–	ND	ND	16.23	NA	NA	NA	NA	NA	ND	–	VUS: PM2, PP3	No	Syndromic (Noonan syndrome) cryptorchidism (Tartaglia et al., 2002)
6	*WDR11*	10:122668121	snv/missense	NM_018117.11:c.3571G>A:p.(Gly1191Ser)	rs149486212	0.0001	0.004	34	14	5	NA	NA	VUS	ND	–	VUS : PP2, PP3	No	Hypospadias (ᴔEggers et al., 2016, Fan et al., 2017), small testes (ᴔFan et al., 2017)
7	*EVC*	4:5800455	snv/missense	NM_153717.2:c.2240C>T:p.(Ala747Val)	rs151091776	0.0002	ND	18.37	5	4	0	NA	VUS	ND	–	VUS: BP4	No	Syndromic (Ellis–van Creveld syndrome) hypospadias (D’Asdia et al., 2013) and micropenis (Ibarra-Ramirez et al., 2017), gonadal development? (Beverdam and Koopman, 2006; Jameson et al., 2012)
7	*MAML3*	4:140811709	snv/missense	NM_018717.5:c.881A>G:p.(Asn294Ser)	rs115966590	0.0028	ND	13.26	7	6	NA	NA	VUS	ND	–	VUS: -	No	Female gonadal development? (Jameson et al., 2012)
7	*NOTCH2*	1:120458982	snv/missense	NM_024408.3:c.6363G>C:p.(Lys2121Asn)	rs144047610	0.0004	0.002	23.4	9	5	NA	NA	VUS	VUS	VUS	VUS: -	DM?, Bicuspid aortic valve	Primary ovarian failure (Patiño et al., 2017); male gonadal development? (Jameson et al., 2012)
7	*PPARGC1B*	5:149219653	snv/missense	NM_133263.3:c.2668G>A:p.(Ala890Thr)	rs150637009	0.0056	ND	19.13	5	5	0	NA	VUS	ND	–	VUS: BP4	No	Candidate to hypospadias (van der Zanden et al., 2012)
7	*WDR11*	10:122637900	snv/missense	NM_018117.11:c.1592C>G:p.(Ser531Cys)	rs775506715	0.00004	ND	24.1	14	6	1	NA	VUS	ND	–	VUS: PP2, PP3	No	Hypospadias (ᴔEggers et al., 2016, Fan et al., 2017), small testes (ᴔFan et al., 2017)
8	*CUL4B*	X:119708447	snv/missense	NM_003588.3:c.26G>A:p.(Gly9Glu)	rs149016283	0.0002	ND	18.73	1	NA	1	NA	Likely benign	Likely benign	Likely benign	Likely benign: BP4, BP6	No	Abnormal genitourinary system (Retterer et al., 2016)
8	*DAPK1*	9:90321476	snv/missense	NM_004938.3:c.3490G>A:p.(Asp1164Asn)	rs937952689	0.00007	ND	24.3	8	5	NA	NA	VUS	ND	–	VUS: -	No	Female gonadal development? (Jameson et al., 2012)
8	*EMX2*	10:119305133	snv/intronic	NM_004098.3:c.407-10C >T	–	0.000004	ND	9.098	NA	NA	1	4	ND	ND	–	VUS: BP4	No	46,XX DSD (Liu et al., 2015), sex determination (Biason-Lauber, 2010; Jakob and Lovell-Badge, 2011; Eggers and Sinclair, 2012), (female) gonadal development (Rey et al., 2000; Jameson et al., 2012; Grinspon and Rey, 2014), (Jameson et al., 2012); 46,XY DSD (Piard et al., 2014)
8	*FREM2*	13:39454885	insertion/frameshift	NM_207361.5:c.9472dupC:p.(Gln3160Thrfs*6)	–	ND	ND	–	NA	NA	NA	NA	ND	ND	–	Likely pathogenic: PVS1, PM2	No	Syndromic (Fraser syndrome) abnormal genitalia (De Bernardo et al., 2015), female gonadal development (Jameson et al., 2012)
8	*IGFBP2*	2:217526593	snv/missense	NM_000597.3:c.685C>A:p.(Gln229Lys)	–	ND	ND	23.7	8	6	0	NA	VUS	ND	–	VUS: PM2, PP3	No entry for this gene	Candidate gene in ovary development (Clement et al., 2007), female gonadal development? (Jameson et al., 2012; Munger et al., 2013).
8	*MAML2*	11:95826575	snv/missense	NM_032427.3:c.620G>A:p.(Arg207His)	rs191391876	0.0002	ND	24.4	9	5	0	NA	VUS	ND	–	VUS: -	No	No (MAMLD1-related)
8	*MAML3*	4:140811709	snv/missense	NM_018717.5:c.881A>G:p.(Asn294Ser)	rs115966590	0.0028	0.004	13.26	7	6	NA	NA	VUS	ND	–	VUS: -	No	Female gonadal development? (Jameson et al., 2012)
8	*MYO7A*	11:76883787	delins/intronic	NM_000260:c.1798-7_1798-6delCCinsAT	–	ND	ND	–	NA	NA	NA	NA	ND	ND	–	–	No	Male gonadal development? (Jameson et al., 2012; Li et al., 2014)
8	*MYO7A*	11:76883790	delins/intronic-exonic	NM_000260: c.1798-4_1801delinsGGCTGCT	–	ND	ND	–	NA	NA	NA	NA	ND	ND	–	–	No	Male gonadal development? (Jameson et al., 2012; Li et al., 2014)
8	*NOTCH1*	9:139405111	snv/missense	NM_017617.5:c.2734C>T:p.(Arg912Trp)	rs201620358	0.002	ND	31	12	6	0	3	Likely benign	VUS	VUS	VUS: PP3	No	Related to SHH and FGF10 (Grinspon and Rey, 2014)
8	*PIK3R3*	1:46521570	snv/missense	NM_003629.3:c.838G>A:p.(Asp280Asn)	rs186728731	0.0001	ND	25.5	6	6	0	2	VUS	ND	–	VUS: PP3	No	Female gonadal development? (Beverdam and Koopman, 2006; Jameson et al., 2012)
8	*TGFBI*	5:135398870	snv/intronic	NM_000358.2:c.2012-5T>C	rs147650812	0.004	0.007	4.531	NA	NA	0	4	ND	ND	–	VUS: -	No	Gonadal development? (Jameson et al., 2012, Clement et al., 2007, Beverdam and Koopman, 2006)
8	*WNT9A*	1:228109247	snv/missense	NM_003395.3:c.1070G>A:p.(Arg357His)	rs145836311	0.0010	0.004	34	11	5	NA	NA	VUS	ND	–	VUS: -	No	Female gonadal development? (Nef et al., 2005; Beverdam and Koopman, 2006)
8	*WNT9B*	17:44949939	snv/missense	NM_003396.2:c.134C>T:p.(Pro45Leu)	rs530502749	0.00002	ND	21.1	5	4	0	NA	VUS	ND	–	VUS: PM2	No	Mayer–Rokitansky–Küster–Hauser syndrome (Waschk et al., 2016); bicornuate uterus (Waschk et al., 2016), organogenesis urogenital system (Carroll et al., 2005; Grinspon and Rey, 2014)

All patients were heterozygote for these variants and were checked and confirmed through IGV software (alignment with human genome hg19/grch37; https://www.broadinstitute.org/igv/, Broad Institute, Cambridge, MA, USA). (1) Combined exonic predictor CADD: Combined Annotation Dependent Depletion (cutoff score: consensus ≥20; deleterious for DSD ≥18; contained in ANNOVAR). (2) Exonic predictors (funcional impact) (ANNOVAR, 15): SIFT, PolyPhen2 HumDiv, PolyPhen2 HumVar, LRT, MutationTaster, MutationAssessor, FATHEMM, Fathmm-MKL, PROVEAN, VEST3 (Variant Effect Scoring Tool), MetaSVM, MetaLR, MCAP, DANN, fitCons. (3) Exonic predictors (evolutionary conservation) (ANNOVAR, 6): GERP++, phyloP (vertebrate and mammalians), phastCons (vertebrate and mammalians), SiPhy. (4) Splicing predictors (ANNOVAR, 3): splicing predictor from dbscSNV ADA and RF, and SPIDEX splicing predictor (DPSI). (5) Splicing predictors (Alamut software, 5): SSF, MaxEnt, NNSPLICE, GeneSplicer, Ex-Skip. (6) InterVar: Clinical Interpretation of genetic Variants by ACMG/AMP guideline (http://wintervar.wglab.org/); ClinVar (https://www.ncbi.nlm.nih.gov/clinvar/); VarSome: The Human Genomics Search Engine (https://varsome.com/); ACMG: ACMG classification from VarSome search engine. (7) Human Gene Mutation Database Biobase (HGMD^®^ Professional 2018.2, http://www.biobase-international.com/product/hgmd): reported variant: variant class, reference, clinical association Human Gene Mutation Database Biobase. (8) Gene characteristics: evidences for genotype-phenotype correlation. Data obtained from pubmed, HGMD and String. ND, not detected; -, not shown; NA, not analysed; no, not previously related to DSD.

We identified a total of 55 potentially deleterious/candidate heterozygous/hemizygous variants in 41 genes in the eight hemizygous/heterozygous *MAMLD1* patients (**Tables 1** and **2**). In the seven 46,XY patients 1–16 variants were found in a total of 1–16 genes, while the 46,XX *MAMLD1* patient revealed 14 additional variants in 13 genes. (**Tables 1** and **2**).

Patient 1 harbored nine variants in seven genes: *CYP1A1*, *EVC*, *GRID1*, *NOTCH1*, *RET*, *RIPK4* and *ZBTB16*, all of them associated to gonadal/genital anomalies (**Table 2**). Patient 2 carried one variant in *RECQL4*, associated with syndromic hypospadias (**Table 2**). Patient 3 presented two variants in two genes: *GLI2* (associated to gonadal/genital anomalies) and *RECQL4* (associated to syndromic hypospadias) (**Table 2**). Patient 4 had four variants in four genes: *CDH23*, *COL9A3*, *MAML1* and *NOTCH1*; all, except *MAML1*, have been proposed to be associated with gonadal development (**Table 2**). In patient 5, six variants in six genes were found: *BNC2*, *FGF10*, *HSD3B2*, *IRX5*, *MAML2* and *NOTCH2*; all, except *MAML2*, have also been associated with hypospadias or gonadal development (**Table 2**). Patient 6 carried 16 variants in 16 genes: *ATF3*, *BNC2*, *CYP1A1*, *EYA1*, *FLNA*, *FRAS1*, *GLI3*, *HOXA13*, *IRX5*, *IRX6*, *MAML1*, *NRP1*, *MAML3, PROP1, PTPN11* and *WDR11* (**Table 2**). Thirteen of these genes are associated with risk of hypospadias and/or syndromes that include abnormal gonadal/genital development, whereas *MAML1* is unrelated, *MAML3* has been proposed to be associated with female gonadal development and *PROP1* has only been associated with anterior pituitary insufficiency/hypogonadotropic hypogonadism. In addition, six of these genes have previously been described in patients with aortic diseases and cardiopathies (**Table 2**). Patient 7 presented five variants in five genes: *EVC*, *MAML3, NOTCH2*, *PPARGC1B* and *WDR11*; four of them associated with hypospadias or male gonadal development and one, *MAML3*, with female gonadal development (**Table 2**). Finally, patient 8 harbored 14 variants in 13 genes: *CUL4B*, *DAPK1*, *EMX2*, *FREM2*, *IGFBP2*, *MAML2*, *MAML3*, *MYO7A*, *NOTCH1*, *PIK3R3*, *TGFBI*, *WNT9A* and *WNT9B*. Among them, only *MAML2* has not been related to gonadal or genitourinary system development (**Table 2**).

The following genes showed variants in two patients: *CYP1A1* in patients 1 and 6; *EVC* in patients 1 (2 variants) and 7; *IRX5* in patients 5 and 6; *MAML1* in patients 4 and 6; *MAML2* in patients 5 and 8; *NOTCH2* in patients 5 and 7; *RECQL4* in patients 2 and 3 and *WDR11* in patients 6 and 7 (**Table 2**). In addition, 2 genes presented variants in 3 patients: *MAML3* (patients 6, 7 and 8) and *NOTCH1* (patients 1, 4 and 8). Furthermore, *RIPK4* presented 2 variants in patient 1. Finally, *BNC2* variant c.1868C>A:p.(Pro623His) (MAF = 0.002) was detected in 2 patients (patient 1 and 7) and *MAML3* variant c.881A>G:p.(Asn294Ser) (MAF = 0.0028) in patients 7 and 8 (**Table 2**).

We performed interactome analysis for the identified DSD genes using bioinformatic tools for the analysis of possible gene-protein interactions. The network comprising all genes identified is shown in **Figure 1**. Overall, a connection was found for 27 of the 41 genes. MAMLD1 connects directly to MAML1/2/3. Via NOTCH1/2 8 genes are in connection with MAMLD1, namely WNT9A/9B, GLI2/3, FGF10, RET, PROP1 and NRP1. Some of these genes are also central nodes for further connections; e.g. GLI3 for EVC, FGF10, GLI2, RIPK4 and EYA1; and RET for PIK3R3 with PTPN11, which also is connected with RIPK4. RIPK4 itself is a central node for ZBTB16, CUL4B, GLI3 and PTPN11. NRP1 is connected to FLNA and EYA1 connects with FRAS1 and FREM2. In addition, 2 isolated gene couples have been revealed by our analysis: CYP1A1-HSD3B2 and MYO7A-CDH23. These observations give an idea of the complex interactions among genes related to sex development.

The specific interactome of identified genes in patients 1 and 4 to 8 is shown in **Figure 2**. In patients 1, 4, 5, 7 and 8, MAMLD1 and MAMLD1-related genes (MAML1, MAML2 or MAML3) are directly related to NOTCH1/2 (**Figures 2A–C, E, F**). In patient 1, there are 2 networks: ZBTB16-RIPK4 and MAMLD1-NOTCH1-RET (**Figure 2A**). In patient 6, GLI3, EYA1 and FRAS1 as well as FLNA and NRP1 seem directly related (**Figure 2D**). In patient 8, NOTCH1 plays a central role connecting to WNT9A, WNT9B and MAMLD1 network (**Figure 2F**).

## Discussion

Sex development is a very complex biological event which requires the concerted collaboration of a large network of genes in a spatial and temporal correct fashion. In the past, much has been learned about human sex development from monogenic DSD, but the broad spectrum of phenotypes in numerous DSD individuals remains a conundrum. Oligogenic disease has been proposed. In fact, multiple genetic hits, which might not be deleterious by themselves, have been found in several individuals with DSD (Kon et al., 2015; Eggers et al., 2016; Mazen et al., 2016; Werner et al., 2017; Camats et al., 2018). In a previous study of 46,XY DSD patients carrying *MAMLD1* variants, we showed that none of the variants were functionally pathogenic except for a stop variant (Camats et al., 2015). In the present study, we searched for additional genetic hits in DSD patients harboring *MAMLD1* mutations and manifesting with unexplained broad phenotypes. Using HTS and a custom-made algorithm including DSD- and *MAMLD1*-related genes from literature and databases, we identified potentially deleterious genetic variants in additional genes in all *MAMLD1* individuals. Thus, we believe that the broad phenotype of individuals carrying *MAMLD1* variants is due to additional genetic hits.

In our study, we identified 55 additional heterozygous/hemizygous variants in 41 genes in seven 46,XY DSD hemizygous and one 46,XX DSD heterozygous *MAMLD1* patients. Among the 41 genes, 16 have been previously reported in humans with hypospadias (*ATF3, BNC2, CYP1A1, EMX2, EYA1, FLNA, GLI3, GRID1, GLI2,, FGF10, HOXA13, HSD3B2, IRX5, IRX6*, *PPARGC1B* and *WDR11* (**Table 2**); 8 have been related to cryptorchidism (*BNC2*, *FLNA, RET, RECQL4, NRP1, PTPN11, RIPK4* and *ZBTB16*), and 5 genes have been found in patients with micropenis (*BNC2, EVC, FGF10, RIPK4 and ZBTB16*). Also, 15 genes have been described in other types of DSD (*CUL4B, EMX2, FRAS1, FREM2, HSD3B2, NOTCH2* and *NRP1*) (**Table 2**) and/or were reported in different syndromes (*CYP1A1, EVC, FRAS1, HOXA13, PTPN11, RECQL4, RET, RIPK4* and *ZBTB16*) (**Table 2**). In addition, 27 genes had been previously described in the context of sex or gonadal development (*ATF3, BNC2, CDH23, COL9A3, DAPK1, EMX2, EVC, EYA1, FLNA, FRAS1, FREM2, GLI2, GLI3, HOXA13, IGFBP2, IRX5, MAML3, MYO7A, NOTCH1, NOTCH2, NRP1, PIK3R3, RET, RIPK4, TGFBI, WNT9A* and *WNT9B*). Thirteen of these genes have been found involved in female gonadal development and 46,XX DSD (*ATF3, DAPK1, EMX2, FLNA, FRAS1, FREM2, GLI3, IGFBP2, IRX5, MAML3, PIK3R3*, *WNT9A* and *WNT9B*), 8 of which in patient 8 (**Table 2**).

According to OMIM, almost all of our patients presented at least one variant in a gene with autosomal dominant inheritance (AD) (*COL9A3, GLI2, FGF10, FLNA, EYA1, GLI3, HOXA13, NOTCH1, NOTCH2, PTPN11, RET, TGFB* and *WDR11*), while other genes (*CDH23, MYO7A* and *PPARGC1B*) may have both AD and autosomal recessive (AR) inheritance. *FLNA* and *CUL4B* are X-linked (XLR), while *CYP1A1, FREM2, EVC, HSD3B2, IRX5, PROP1*, *RAS1, RECQL4*, *RIPK4* and *ZBTB16* are known for AR inheritance. No information on inheritance is currently available for the remaining genes including *ATF3, BNC2, GRID1, DAPK1, IRX6, IGFBP2, MAML1, MAML2, MAML3, NRP1, PIK3R3, WNT9A* and *WNT9B*.

The seven *MAMLD1* patients with 46,XY DSD presented phenotypes from female external genitalia (patient 4) to variable degrees of hypospadias, cryptorchidism and small penis (**Table 1**). Interestingly, patient 4 with female external genitalia had normal T secretion. Similarly, patient 5 carrying a heterozygous *HSD3B2* variant, had normal levels of 17OH-pregnenolone, DHEA and DHEA-S (data not shown). Patient 6, who presented with a right aortic arch, was found to carry variants in five genes (*BNC2, FLNA, MAML1, NRP1* and *PTPN11*) that have been previously described in patients with heart and/or vascular anomalies (Tartaglia et al., 2002; Bhoj et al., 2011; Lee et al., 2014; Shaheen et al., 2015; Preuss et al., 2016; Chen et al., 2018). The 46,XX patient (patient 8), with primary amenorrhea, hypergonadotropic hypogonadism, normal female external genitalia and small uterus harbored gene variants involved in gonadal development and DSD (*CUL4B*, *DAPK1*, *EMX2*, *FREM2*, *IGFBP2*, *MAML3*, *MYO7A*, *NOTCH1*, *PIK3R3*, *TGFBI*, *WNT9A* and *WNT9B*; **Table 2**). Five of these genes (*DAPK1, IGFBP2, MAML3, PIK3R3* and *WNT9A*) have so far only been related to female gonadal development (**Table 2**).

Overall, the genes detected in our eight studied patients with *MAMLD1* variants have been previously reported in humans with hypospadias, cryptorchidism, micropenis, and other urogenital abnormalities; or they have been found involved in sexual and gonadal development. Also, some of them have been associated with specific syndromes in patients with genitourinary anomalies: CAKUT syndrome, Ellis–van Creveld syndrome, Fraser syndrome 1, Fraser syndrome 2, hand–foot–genital/Guttmacher syndrome, Noonan syndrome, Mayer–Rokitansky–Küster–Hauser syndrome, Popliteal pterygium syndrome and Rothmund–Thomson syndrome (see **Table 2**). However, none of the present patients presented a complete phenotype for any of these syndromes, maybe because none of the variants completely impairs gene expression and protein function, as inferred by the *in silico* analyses. Detailed information on these genes from current literature is given in **Supplementary Materials (S1)**.

A search for an underlying network comprising variants in the identified genes related to *MAMLD1* revealed a considerable number of genes which showed gene-gene, gene-protein or protein-protein interactions (**Figures 1** and **2**) suggesting that genetic variations in these genes may affect sex development. In addition, *MAML3* was found in a network related to female gonadal development (Jameson et al., 2012). Accordingly, one variant in *MAML3* was present in our 46,XX patient. The analysis of gene/protein network interactions per patient gives an idea of the complexity of the interactions among genes related to sex development. The more variants detected in DSD-related genes, the better to build an interaction network searching for clues on genetic relationship(s) for sex development. In our DSD individuals carrying *MAMLD1* variants, three genes seemed prominent in the network analysis, *NOTCH1, NOTCH2* and *GLI3*. NOTCH signaling is a highly conserved signaling pathway and comprises 4 transmembrane receptors. It is essential for the regulation of embryonic development of multiple organ systems including gonadal development (Windley and Wilhelm, 2016). NOTCH signaling is implicated in Leydig cell differentiation in an inhibitory regulatory fashion (Windley and Wilhelm, 2016). Autosomal dominant mutations in *NOTCH1* cause the Adams–Oliver syndrome (OMIM 616028), while autosomal dominant mutations in *NOTCH2* are reported in the Alagille syndrome 2 (OMIM 610205) and in the Hajdu–Cheney syndrome (OMIM 102500). By contrast, GLI3 is a zinc-finger transcription factor belonging to the desert hedgehog (DHH) signal transduction pathway. DHH signaling is essential for driving Leydig cell differentiation (Windley and Wilhelm, 2016). Thus, NOTCH and DHH signaling work together to regulate Leydig cell development (Windley and Wilhelm, 2016). Autosomal dominant mutations in *GLI3* are described in the Pallister–Hall syndrome (OMIM 146510) or in the Greig cephalopolysyndactyly syndrome (OMIM 175700).

Taken together, our results expand the landscape of genes possibly involved in DSD by revealing both new and old players. Genetic platforms for DSD diagnostics currently consider about 270 genes that have been identified with monogenetic forms of DSD in (mostly) several independent individuals (Cools et al., 2018). Our eight *MAMLD1* individuals share variants in 19 genes comprised in such DSD panels, including *ATF3, BNC2, CUL4B, EVC, FLNA, FRAS1, FREM2, GLI3, HOXA13, HSD3B2, IRX5, NOTCH2, PROP1, PTPN11, RECQL4, RET, RIPK4, WDR11* and *ZBTB16*. By contrast, through our work 22 new genes are now added for considering with differences in sex development: *CDH23, COL9A3, CYP1A1, DAPK1, EMX2, EYA1, FGF10, GLI2, GRID1, IGFBP2, IRX6, MAML1, MAML2, MAML3, MYO7A, NOTCH1, NRP1, PIK3R3, PPARGC1B, TGFBI, WNT9A* and *WNT9B*.

Ideally, genetic variants are tested functionally for proof of their disease-causing effect in model systems. However, when finding multiple variants, which may all contribute only partially, such testing is no longer feasible. Therefore, the likelihood of disease-causing effect of identified variants was assessed in our study by established bioinformatic tools for genetics and by assessing the genotype-phenotype correlation in each patient with current knowledge from literature and databases in the field. In future studies with bigger sample size, next-generation statistical genetic analyses may be employed to identify associations between a group of variants and the complex trait of sex development (Weissenkampen et al., 2019).

In summary, HTS analysis indicates that the broad DSD phenotypes of *MAMLD1* patients may be due to additional variants in other DSD-related genes. We found up to 55 additional genetic hits that may contribute to the DSD phenotype making an oligogenic causation plausible. Bioinformatic network analysis can help in interpreting complex genetic data and put identified single candidate genes into a greater perspective to understand their possible role in DSD biology.

## Data Availability

The datasets generated for this study are publicly available in dbSNP (Sherry, 2001): https://www.ncbi.nlm.nih.gov/projects/SNP/snp_viewBatch.cgi?sbid=1063030.

## Ethics Statement

The study was approved by the Ethics Committee of Hospital Universitari Vall d'Hebron (Barcelona, Spain) (CEIC: PR(IR)23/2016). Written informed consent was obtained from the patients for the publication of their cases.

## Author Contributions

**CF:** Conceptualization, funding acquisition, investigation, methodology, interpretation, project administration, resources, supervision, writing – original draft preparation, **writing – review and editing.** LA: Interpretation, supervision, resources, visualization, **writing – review and editing. MF-C: Investigation**, **writing – review and editing.** K-SS: Validation, interpretation, **writing – review and editing.** IM: Resources, interpretation, **writing – review and editing.** LC: Resources, **writing – review and editing.** IE: Resources, **writing – review and editing.** NC: Conceptualization, data-curation, formal analysis, investigation, methodology, interpretation, project administration, supervision, visualization, writing – original draft preparation, **writing – review and editing.**

## Funding

This work was supported by grants of the Swiss National Science Foundation (http://www.snf.ch) (320030-146127) to CF, the Instituto de Salud Carlos III (www.isciii.es/; Madrid, Spain) Centro de Investigación Biomédica en Red de Enfermedades Raras (CIBERER, http://www.ciberer.es/) U-712 to MF-C, the Agency for Management of University and Research Grants (AGAUR; agaur.gencat.cat), Barcelona, Spain (2009SGR31) to LA, and by the Beatriu de Pinós Fellowship 2014 BP-B 00145 (AGAUR, Catalonia, *Spain*), the Instituto de Salud Carlos III (www.isciii.es/; Madrid, Spain) Centro de Investigación Biomédica en Red de Enfermedades Raras (CIBERER; http://www.ciberer.es/) U-712 to NC.

## Conflict of Interest Statement

The authors declare that the research was conducted in the absence of any commercial or financial relationships that could be construed as a potential conflict of interest.
