# Supplementary material for: Broad Phenotypes of Disorders/Differences of Sex Development in MAMLD1 Patients Through Oligogenic Disease
Source: Front Genet. 2019 Aug 29;10:746. doi: 10.3389/fgene.2019.00746 (PMC6726737; doi:10.3389/fgene.2019.00746)
Supplement: Supplementary file 1 [file DataSheet_1.pdf]

Supplementary Material S1.

Summary of information found in the current published literature for the identified genes suggested contributing to a broad DSD phenotype in 8 individuals with variants in *MAMLD1*

Overall, the genes detected in our 8 studied patients with *MAMLD1* variants have been previously reported in humans with hypospadias, cryptorchidism, small penis, and other urogenital abnormalities. Also, some of them have been associated with specific syndromes (Table 2). The detailed information is summarized below.

- *ATF3* variants were present in patients with hypospadias (Beleza-Meireles et al., 2008; Kalfa et al., 2009; van der Zanden et al., 2012) and this gene is upregulated in prepuce tissue in patients with hypospadias (Wang et al., 2007).
- *BNC2* variants had been related to hypospadias (Baxter et al., 2015; Bhoj et al., 2011; Kon et al., 2015; van der Zanden et al., 2012) and to gonadal development (Jameson et al., 2012). In Bhoj *et al,* the patient also presented with partial anomalous pulmonary venous return (Bhoj et al., 2011).
- Some studies indicate that *CDH23* is an SRY- and SOX9-target (Li et al., 2014) and is overexpressed in male mice gonadal supporting cells (Jameson et al., 2012).
- *COL9A3* is related to male gonadal development (Beverdam and Koopman, 2006; Jameson et al., 2012; Nef et al., 2005).
- *CUL4B* variants were detected in a patient with abnormal genitourinary system (Retterer et al., 2015).
- *CYP1A1* has been related to hypospadias (van der Zanden et al., 2012).
- *FGF10* plays an important role in the morphogenesis of distal genital tubercle structures, like the glans penis (Grinspon and Rey, 2014; Rey et al., 2000) and is linked to increased risk of hypospadias in humans (Carmichael et al., 2013; Svechnikov et al., 2014; van der Zanden et al., 2012).
- *DAPK1* is overexpressed during mice female gonadal development (Jameson et al., 2012).
- An *EVC* mutation has been described in a patient with syndromic (Ellis-van Creveld syndrome) micropenis (D’Asdia et al., 2013; Ibarra-Ramirez et al., 2017) and has also been related to sex development in mice (Beverdam and Koopman, 2006; Jameson et al., 2012).
- *EYA1* is associated to hypospadias (Grinspon and Rey, 2014; Hwang et al., 2014) and one patient with variants presented urinary tract anomalies including hypospadias (Hwang et al., 2014).
- *FLNA* presents high genetic variability. It has been associated to different clinical phenotypes including aortic diseases and cardiopathies (Chen et al., 2018). Two 46,XY brothers were reported with hypospadias, cryptorchidism, testicular atrophy, vaginal duct and diminished nuclear androgen receptor (Carrera-García et al., 2017). It is overexpressed in mice female germ cells during sex development (Jameson et al., 2012).

- *EMX2* variants are present in patients with uterus didelphys (Liu et al., 2015) and in one 46,XY DSD in heterozygosis (Piard et al., 2014). *Emx2* mutant mice lack gonads and genital tracts (Li et al., 2014). *Emx2* expression is necessary for Wolffian and Müllerian duct development in mice (Grinspon and Rey, 2014; Rey et al., 2000), for sex determination (Biason-Lauber, 2010) as it is expressed in early coelomic epithelium in mice (Jakob and Lovell-Badge, 2011; Jameson et al., 2012), and required in the development of the bipotential gonad in mice (Eggers and Sinclair, 2012).
- *FRAS1* is associated to abnormalities in genitourinary system in Fraser syndrome 1 (Kornacki et al., 2017; Retterer et al., 2015).
- *FREM2* is related to Fraser syndrome 2, with congenital genitalia anomalies (De Bernardo et al., 2015) and overexpressed in supporting and stroma cells during mice female gonadal development (Jameson et al., 2012).
- *GLI2* has been associated to hypospadias increased risk (Carmichael et al., 2013), and is considered a growth factor in urogenital development. It participates in masculinization of external genitalia: outgrowth and patterning of external genitalia and urogenital sinus and in the inhibition of apoptosis in penile smooth muscle (Rey et al., 2000).
- *GLI3* has been associated with genitourinary anomalies, hypogonadotropic hypogonadism (Quaynor et al., 2016) and to increased risk of hypospadias (Carmichael et al., 2013). It has been reported to be related to abnormal early morphogenesis of genital primordia (Rey and Grinspon, 2011) and to male gonadal development (Windley and Wilhelm, 2016).
- *HSD3B2* encodes for the steroidogenic enzyme 3-beta-hydroxysteroid dehydrogenase type 2, which is well-known to participate in sex development (Baxter et al., 2015) (Baxter and Vilain, 2013) and steroid hormone synthesis (McCartin et al., 2000). Patients with pathogenic mutations in *HSD3B2* present 46,XY DSD (Codner et al., 2004; Eggers et al., 2016; Kon et al., 2015). Interestingly, although inheritance is mostly AR, some heterozygous patients with hypospadias had been reported (Codner et al., 2004).
- *GRID1* (van der Zanden et al., 2012) has been related to hypospadias.
- *HOXA13* has been associated to hypospadias and to early patterning stage of development (Beleza-Meireles et al., 2007; van der Zanden et al., 2012). It has also been reported in patients with Hand-foot-genital (HFG)/Guttmacher syndrome, which includes hypospadias (Innis et al., 2002), in a patient with HFG and small penis (Goodman et al., 2000) and in another patient with HFG, heart defects & facial anomalies (Jun et al., 2011). It plays a role as growth factor in the development of genital tubercle, accessory sex glands and Wolffian and Müllerian ducts (Grinspon and Rey, 2014; van der Zanden et al., 2012).

- No patients with *IGFBP2* variants have been reported to date. This gene is considered candidate gene in ovary development (Clement et al., 2007) and overexpressed in mice female gonadal development (Jameson et al., 2012; Munger et al., 2013).
- *IRX5* and *IRX6* have been associated to hypospadias in humans (Geller et al., 2014; Grinspon and Rey, 2014).
- *MAML1*, a MAMLD1-related gene, has not been related to DSD yet, but some patients with *MAML1* variants showed cardiomyopathy (left-ventricular outflow tract obstructions) (Preuss et al., 2016).
- *MAML2* (MAMLD1-related) has not been related to DSD to date.
- *MAML3* (MAMLD1-related) is overexpressed during mice female gonadal development (Jameson et al., 2012).
- *MYO7A* is considered SRY- and SOX9-target (Li et al., 2014) and overexpressed in mice male gonadal supporting cells during gonadal development (Jameson et al., 2012).
- *NOTCH1* activity is maintained by FGF10 and SHH to promote prostate growth (Grinspon and Rey, 2014) and it has been related to male gonadal development (Windley and Wilhelm, 2016).
- *NOTCH2* has been related to primary ovarian failure (Patiño et al., 2017) and mice male gonadal development (Jameson et al., 2012; Windley and Wilhelm, 2016).
- *NRP1* has been reported in a female patient with no uterus and absent Fallopian tubes, inguinal testes with calcifications and abnormal histology, deafness and impaired cognition (Baxter et al., 2015) and in another patient with truncus arteriosus, but no DSD (Shaheen et al., 2015). This gene interacts with *Sem3a* (hypogonadotropic hypogonadism and Kallmann syndrome) (Hanchate et al., 2012). It is overexpressed in mice developing gonadal cells (Jameson et al., 2012).
- *PIK3R3* is an SRY target (Li et al., 2014) and related to mice female gonadal development: overexpressed in mice female gonadal cells (Beverdam and Koopman, 2006; Jameson et al., 2012).
- *PPARGC1B* has been considered candidate gene for hypospadias (van der Zanden et al., 2012).
- *PROP1* has been related to human central causes of hypogonadism (combined pituitary hormone deficiency and hypogonadotropic hypogonadism) (Baxter et al., 2015; Baxter and Vilain, 2013; Eggers et al., 2016; Reynaud et al., 2005).
- *PTPN11* presented pathogenic variants in Noonan-syndrome patients with cryptorchidism (Tartaglia et al., 2002) and pulmonic stenosis (Lee et al., 2014; Tartaglia et al., 2002).
- Mutations in *RECQL4* have been detected in a patient with hypospadias and bilateral inguinal hernia (Rothmund-Thomson syndrome) (Kellermayer et al., 2005).
- *RET* deleterious mutations have been detected in syndromic patients with cryptorchidism (CAKUT syndrome) (Chatterjee et al., 2012). In addition, *RET* is overexpressed in germ cells during gonadal development (Jameson et al., 2012) and is a *SRY* target gene in mice (Li et al., 2014).

- *RIPK4* has been related to genital hypoplasia (Kalay et al., 2012; Mitchell et al., 2012), micropenis, hypoplastic scrotum and inguinal hernia (Kalay et al., 2012) in patients with Popliteal Pterygium/Bartsocas-Papas syndrome.
- *TGFBI* is related to gonadal development in both sexes (Beverdam and Koopman, 2006; Clement et al., 2007; Jameson et al., 2012).
- Little is known about *WNT9A*. It seems that it is overexpressed at early mice female gonadal development (Beverdam and Koopman, 2006; Nef et al., 2005).
- *WNT9B* variants have been found in Mayer-Rokitansky-Küster-Hauser syndrome (no uterus, no vagina) (Waschk et al., 2016), in one patient with congenital anomalies of the kidney and urinary tract (Nicolaou et al., 2016) and in another patient with bicornuate uterus (Waschk et al., 2016). WNT9B is secreted by Wolffian ducts to inhibit elongation of Müllerian ducts (Grinspon and Rey, 2014) and is believed to be involved in mesenchymal to epithelial transition in organogenesis of mammalian urogenital system (Carroll et al., 2005; Grinspon and Rey, 2014).
- Variants in *WDR11* have been detected in patients with 46,XY DSD (Fan et al., 2017), patients with hypospadias (Eggers et al., 2016; Fan et al., 2017), small testes (Fan et al., 2017) and with hypogonadotropic hypogonadism (Dong et al., 2016; Eggers et al., 2016).
- *ZBTB16* mutations have been reported in a syndromic patient (deletion 11q23) with cryptorchidism and micropenis (Fischer et al., 2008).

REFERENCES

Baxter, R. M., Arboleda, V. A., Lee, H., Barseghyan, H., Adam, M. P., Fechner, P. Y., et al. (2015). Exome Sequencing for the Diagnosis of 46,XY Disorders of Sex Development. *J. Clin. Endocrinol. Metab.* 100, E333–E344. doi:10.1210/jc.2014-2605.

Baxter, R. M., and Vilain, E. (2013). Translational genetics for diagnosis of human disorders of sex development. *Annu Rev Genomics Hum Genet* 14, 371–392. doi:10.1146/annurev-genom-091212-153417.

Beleza-Meireles, A., Lundberg, F., Lagerstedt, K., Zhou, X., Omrani, D., Frisen, L., et al. (2007). FGFR2, FGF8, FGF10 and BMP7 as candidate genes for hypospadias. *Eur J Hum Genet* 15, 405–410. doi:5201777 [pii]10.1038/sj.ejhg.5201777.

Beleza-Meireles, A., Töhönen, V., Söderhäll, C., Schwentner, C., Radmayr, C., Kockum, I., et al. (2008). Activating transcription factor 3: A hormone responsive gene in the etiology of hypospadias. *Eur. J.*

Endocrinol. doi:10.1530/EJE-07-0793.

Beverdam, A., and Koopman, P. (2006). Expression profiling of purified mouse gonadal somatic cells during the critical time window of sex determination reveals novel candidate genes for human sexual dysgenesis syndromes. *Hum Mol Genet* 15, 417–431. doi:ddi463 [pii]10.1093/hmg/ddi463.

Bhoj, E. J., Ramos, P., Baker, L. A., Cost, N., Nordenskjöld, A., Elder, F. F., et al. (2011). Human balanced translocation and mouse gene inactivation implicate Basonuclin 2 in distal urethral development. *Eur. J. Hum. Genet.* doi:10.1038/ejhg.2010.245.

Biason-Lauber, A. (2010). Control of sex development. *Best Pr. Res Clin Endocrinol Metab* 24, 163–186. doi:10.1016/j.beem.2009.12.002.

Carmichael, S. L., Ma, C., Choudhry, S., Lammer, E. J., Witte, J. S., and Shaw, G. M. (2013). Hypospadias and genes related to genital tubercle and early urethral development. *J Urol* 190, 1884–1892. doi:10.1016/j.juro.2013.05.061S0022-5347(13)04514-X [pii].

Carrera-García, L., Rivas-Crespo, M. F., and Fernández García, M. S. (2017). Androgen receptor dysfunction as a prevalent manifestation in young male carriers of a FLNA gene mutation. *Am. J. Med. Genet. Part A.* doi:10.1002/ajmg.a.38230.

Carroll, T. J., Park, J. S., Hayashi, S., Majumdar, A., and McMahon, A. P. (2005). Wnt9b plays a central role in the regulation of mesenchymal to epithelial transitions underlying organogenesis of the mammalian urogenital system. *Dev. Cell*. doi:10.1016/j.devcel.2005.05.016.

Chatterjee, R., Ramos, E., Hoffman, M., Vanwinkle, J., Martin, D. R., Davis, T. K., et al. (2012). Traditional and targeted exome sequencing reveals common, rare and novel functional deleterious variants in RET-signaling complex in a cohort of living US patients with urinary tract malformations. *Hum. Genet.* doi:10.1007/s00439-012-1181-3.

Chen, M. H., Choudhury, S., Hirata, M., Khalsa, S., Chang, B., and Walsh, C. A. (2018). Thoracic aortic aneurysm in patients with loss of function Filamin A mutations: Clinical characterization, genetics, and recommendations. *Am. J. Med. Genet. Part A.* doi:10.1002/ajmg.a.38580.

Clement, T. M., Anway, M. D., Uzumcu, M., and Skinner, M. K. (2007). Regulation of the gonadal transcriptome during sex determination and testis morphogenesis: comparative candidate genes. *Reproduction* 134, 455–472. doi:134/3/455 [pii]10.1530/REP-06-0341.

Codner, E., Okuma, C., Iñiguez, G., Boric, M. A., Avila, A., Johnson, M. C., et al. (2004). Molecular Study of the 3β-Hydroxysteroid Dehydrogenase Gene Type II in Patients with Hypospadias. *J. Clin.*

Endocrinol. Metab. doi:10.1210/jc.2002-020873.

D'Asdia, M. C., Torrente, I., Consoli, F., Ferese, R., Magliozzi, M., Bernardini, L., et al. (2013). Novel and recurrent EVC and EVC2 mutations in Ellis-van Creveld syndrome and Weyers acrofacial dyostosis. *Eur. J. Med. Genet.* doi:10.1016/j.ejmg.2012.11.005.

De Bernardo, G., Giordano, M., Di Toro, A., Sordino, D., and De Brasi, D. (2015). Prenatal diagnosis of Fraser syndrome: a matter of life or death? *Ital. J. Pediatr.* doi:10.1186/s13052-015-0195-6.

Dong, Y., Yi, Y., Yao, H., Yang, Z., Hu, H., Liu, J., et al. (2016). Targeted next-generation sequencing identification of mutations in patients with disorders of sex development. *BMC Med Genet* 17, 23. doi:10.1186/s12881-016-0286-2.

Eggers, S., Sadedin, S., van den Bergen, J. A., Robevska, G., Ohnesorg, T., Hewitt, J., et al. (2016). Disorders of sex development: Insights from targeted gene sequencing of a large international patient cohort. *Genome Biol.* 17. doi:10.1186/s13059-016-1105-y.

Eggers, S., and Sinclair, A. (2012). Mammalian sex determination-insights from humans and mice. *Chromosom. Res.* doi:10.1007/s10577-012-9274-3.

Fan, Y., Zhang, X., Wang, L., Wang, R., Huang, Z., Sun, Y., et al. (2017). Diagnostic application of targeted next-generation sequencing of 80 genes associated with disorders of sexual development. *Sci. Rep.* 7. doi:10.1038/srep44536.

Fischer, S., Kohlhase, J., Böhm, D., Schweiger, B., Hoffmann, D., Heitmann, M., et al. (2008). Biallelic loss of function of the promyelocytic leukaemia zinc finger (PLZF) gene causes severe skeletal defects and genital hypoplasia. *J. Med. Genet.* doi:10.1136/jmg.2008.059451.

Geller, F., Feenstra, B., Carstensen, L., Pers, T. H., Van Rooij, I. A. L. M., Körberg, I. B., et al. (2014). Genome-wide association analyses identify variants in developmental genes associated with hypospadias. *Nat. Genet.* 46. doi:10.1038/ng.3063.

Goodman, F. R., Bacchelli, C., Brady, A. F., Brueton, L. A., Fryns, J.-P., Mortlock, D. P., et al. (2000). Novel HOXA13 Mutations and the Phenotypic Spectrum of Hand-Foot-Genital Syndrome. *Am. J. Hum. Genet.* doi:10.1086/302961.

Grinspon, R. P., and Rey, R. A. (2014). When hormone defects cannot explain it: Malformative disorders of sex development. *Birth Defects Res. Part C - Embryo Today Rev.* 102, 359–373. doi:10.1002/bdrc.21086.

Hanchate, N. K., Giacobini, P., Lhuillier, P., Parkash, J., Espy, C., Fouveaut, C., et al. (2012). SEMA3A, a Gene Involved in Axonal Pathfinding, Is Mutated in Patients with Kallmann Syndrome. *PLoS Genet.* doi:10.1371/journal.pgen.1002896.

Hwang, D. Y., Dworschak, G. C., Kohl, S., Saisawat, P., Vivante, A., Hilger, A. C., et al. (2014). Mutations in 12 known dominant disease-causing genes clarify many congenital anomalies of the kidney and urinary tract. *Kidney Int.* doi:10.1038/ki.2013.508.

Ibarra-Ramirez, M., Campos-Acevedo, L. D., Lugo-Trampe, J., Martínez-Garza, L. E., Martinez-Glez, V., Valencia-Benitez, M., et al. (2017). Phenotypic Variation in Patients with Homozygous c.1678G>T Mutation in EVC Gene: Report of Two Mexican Families with Ellis-van Creveld Syndrome. *Am. J. Case Rep.* 18, 1325–1329.

Innis, J. W., Goodman, F. R., Bacchelli, C., Williams, T. M., Mortlock, D. P., Sateesh, P., et al. (2002). A HOXA13 allele with a missense mutation in the homeobox and a dinucleotide deletion in the promoter underlies Guttmacher syndrome. *Hum. Mutat.* doi:10.1002/humu.9036.

Jakob, S., and Lovell-Badge, R. (2011). Sex determination and the control of Sox9 expression in mammals. *FEBS J* 278, 1002–1009. doi:10.1111/j.1742-4658.2011.08029.x.

Jameson, S. A., Natarajan, A., Cool, J., DeFalco, T., Maatouk, D. M., Mork, L., et al. (2012). Temporal transcriptional profiling of somatic and germ cells reveals biased lineage priming of sexual fate in the fetal mouse gonad. *PLoS Genet* 8, e1002575. doi:10.1371/journal.pgen.1002575PGENETICS-D-11-02591 [pii].

Jun, K. R., Seo, E. J., Lee, J. O., Yoo, H. W., Park, I. S., and Yoon, H. K. (2011). Molecular cytogenetic and clinical characterization of a patient with a 5.6-Mb deletion in 7p15 including HOXA cluster. *Am. J. Med. Genet. Part A.* doi:10.1002/ajmg.a.33860.

Kalay, E., Sezgin, O., Chellappa, V., Mutlu, M., Morsy, H., Kayserili, H., et al. (2012). Mutations in RIPK4 cause the autosomal-recessive form of popliteal pterygium syndrome. *Am. J. Hum. Genet.* doi:10.1016/j.ajhg.2011.11.014.

Kalfa, N., Philibert, P., and Sultan, C. (2009). Is hypospadias a genetic, endocrine or environmental disease, or still an unexplained malformation? *Int. J. Androl.* doi:10.1111/j.1365-2605.2008.00899.x.

Kellermayer, R., Sütonen, H. A., Hadzsiev, K., Kestilä, M., and Kosztolányi, G. (2005). A patient with Rothmund-Thomson syndrome and all features of RAPADILINO. *Arch. Dermatol.* doi:10.1001/archderm.141.5.617.

Kon, M., Suzuki, E., Dung, V. C., Hasegawa, Y., Mitsui, T., Muroya, K., et al. (2015). Molecular basis of non-syndromic hypospadias: Systematic mutation screening and genome-wide copy-number analysis of 62 patients. *Hum. Reprod.* 30, 499–506. doi:10.1093/humrep/deu364.

Kornacki, J., Sowinska-Seidler, A., Socha, M., Ropacka, M., and Jamsheer, A. (2017). Prenatal diagnosis of Fraser syndrome using routine ultrasound examination, confirmed by exome sequencing: Report of a novel homozygous missense FRAS1 mutation. *Congenit. Anom. (Kyoto).* 57, 37–38.

Lee, H., Deignan, J. L., Dorrani, N., Strom, S. P., Kantarci, S., Quintero-Rivera, F., et al. (2014). Clinical exome sequencing for genetic identification of rare Mendelian disorders. *JAMA* 312, 1880–1887. doi:10.1001/jama.2014.146041918775 [pii].

Li, Y., Zheng, M., and Lau, Y. F. (2014). The sex-determining factors SRY and SOX9 regulate similar target genes and promote testis cord formation during testicular differentiation. *Cell Rep* 8, 723–733. doi:10.1016/j.celrep.2014.06.055S2211-1247(14)00557-9 [pii].

Liu, S., Gao, X., Qin, Y., Liu, W., Huang, T., Ma, J., et al. (2015). Nonsense mutation of EMX2 is potential causative for uterus didelphysis: First molecular explanation for isolated incomplete müllerian fusion. *Fertil. Steril.* doi:10.1016/j.fertnstert.2014.11.030.

McCartin, S., Russell, A. J., Fisher, R. A., Wallace, A. M., Arnhold, I. J. P., Mason, J. I., et al. (2000). Phenotypic variability and origins of mutations in the gene encoding 3β-hydroxysteroid dehydrogenase type II. *J. Mol. Endocrinol.* doi:10.1677/jme.0.0240075.

Mitchell, K., O'Sullivan, J., Missero, C., Blair, E., Richardson, R., Anderson, B., et al. (2012). Exome sequence identifies RIPK4 as the Bartsocas-Papas syndrome locus. *Am. J. Hum. Genet.* doi:10.1016/j.ajhg.2011.11.013.

Munger, S. C., Natarajan, A., Looger, L. L., Ohler, U., and Capel, B. (2013). Fine time course expression analysis identifies cascades of activation and repression and maps a putative regulator of mammalian sex determination. *PLoS Genet* 9, e1003630. doi:10.1371/journal.pgen.1003630PGENETICS-D-13-00601 [pii].

Nef, S., Schaad, O., Stallings, N. R., Cederroth, C. R., Pitetti, J. L., Schaer, G., et al. (2005). Gene expression during sex determination reveals a robust female genetic program at the onset of ovarian development. *Dev Biol* 287, 361–377. doi:S0012-1606(05)00602-0 [pii]10.1016/j.ydbio.2005.09.008.

Nicolaou, N., Pulit, S. L., Nijman, I. J., Monroe, G. R., Feitz, W. F. J., Schreuder, M. F., et al. (2016). Prioritization and burden analysis of rare variants in 208 candidate genes suggest they do not play a

major role in CAKUT. *Kidney Int.* doi:10.1038/ki.2015.319.

Patiño, L. C., Beau, I., Carlosama, C., Buitrago, J. C., González, R., Suárez, C. F., et al. (2017). New mutations in non-syndromic primary ovarian insufficiency patients identified via whole-exome sequencing. *Hum. Reprod.* doi:10.1093/humrep/dex089.

Piard, J., Mignot, B., Arbez-Gindre, F., Aubert, D., Morel, Y., Roze, V., et al. (2014). Severe sex differentiation disorder in a boy with a 3.8 Mb 10q25.3-q26.12 microdeletion encompassing EMX2. *Am. J. Med. Genet. Part A.* doi:10.1002/ajmg.a.36662.

Preuss, C., Capredon, M., Wünnemann, F., Chetaille, P., Prince, A., Godard, B., et al. (2016). Family Based Whole Exome Sequencing Reveals the Multifaceted Role of Notch Signaling in Congenital Heart Disease. *PLoS Genet.* doi:10.1371/journal.pgen.1006335.

Quaynor, S. D., Bosley, M. E., Duckworth, C. G., Porter, K. R., Kim, S. H., Kim, H. G., et al. (2016). Targeted next generation sequencing approach identifies eighteen new candidate genes in normosmic hypogonadotropic hypogonadism and Kallmann syndrome. *Mol. Cell. Endocrinol.* doi:10.1016/j.mce.2016.08.007.

Retterer, K., Juusola, J., Cho, M. T., Vitazka, P., Millan, F., Gibellini, F., et al. (2015). Clinical application of whole-exome sequencing across clinical indications. *Genet. Med.* doi:10.1038/gim.2015.148.

Rey, R. A., and Grinspon, R. P. (2011). Normal male sexual differentiation and aetiology of disorders of sex development. *Best Pr. Res Clin Endocrinol Metab* 25, 221–238. doi:10.1016/j.beem.2010.08.013S1521-690X(10)00113-2 [pii].

Rey, R., Josso, N., and Racine, C. (2000). "Sexual Differentiation," in *Endotext*, eds. L. J. De Groot, G. Chrousos, K. Dungan, K. R. Feingold, A. Grossman, J. M. Hershman, et al. (South Dartmouth (MA)). Available at: http://www.ncbi.nlm.nih.gov/pubmed/25905232.

Reynaud, R., Barlier, A., Vallette-Kasic, S., Saveanu, A., Guillet, M. P., Simonin, G., et al. (2005). An uncommon phenotype with familial central hypogonadism caused by a novel PROP1 gene mutant truncated in the transactivation domain. *J. Clin. Endocrinol. Metab.* doi:10.1210/jc.2005-0119.

Shaheen, R., Hashem, A. Al, Alghamdi, M. H., Seidahmad, M. Z., Wakil, S. M., Dagriri, K., et al. (2015). Positional mapping of PRKD1, NRP1 and PRDM1 as novel candidate disease genes in truncus arteriosus. *J. Med. Genet.* doi:10.1136/jmedgenet-2015-102992.

Svechnikov, K., Stukenborg, J. B., Savchuck, I., and Soder, O. (2014). Similar causes of various reproductive disorders in early life. *Asian J Androl* 16, 50–59. doi:10.4103/1008-

682X.122199AsianJAndrol_2014_16_1_50_122199 [pii].

Tartaglia, M., Kalidas, K., Shaw, A., Song, X., Musat, D. L., Burgt, I. Van Der, et al. (2002). PTPN11 Mutations in Noonan Syndrome : Molecular Spectrum , Genotype-Phenotype Correlation , and Phenotypic Heterogeneity. *Am J Hum Genet*. doi:10.1086/340847.

van der Zanden, L. F., van Rooij, I. A., Feitz, W. F., Franke, B., Knoers, N. V, and Roeleveld, N. (2012). Aetiology of hypospadias: a systematic review of genes and environment. *Hum Reprod Updat.* 18, 260–283. doi:10.1093/humupd/dms002dms002 [pii].

Wang, Z., Liu, B. C., Lin, G. T., Lin, C. S., Lue, T. F., Willingham, E., et al. (2007). Up-Regulation of Estrogen Responsive Genes in Hypospadias: Microarray Analysis. *J. Urol.* doi:10.1016/j.juro.2007.01.014.

Waschk, D. E. J., Tewes, A. C., Römer, T., Hucke, J., Kapczuk, K., Schippert, C., et al. (2016). Mutations in WNT9B are associated with Mayer-Rokitansky-Küster-Hauser syndrome. *Clin. Genet.* doi:10.1111/cge.12701.

Windley, S. P., and Wilhelm, D. (2016). Signaling Pathways Involved in Mammalian Sex Determination and Gonad Development. *Sex. Dev.* doi:10.1159/000444065.
